# Vowel segmentation impact on machine learning classification for chronic obstructive pulmonary disease

**DOI:** 10.1038/s41598-025-95320-3

**Published:** 2025-03-22

**Authors:** Alper Idrisoglu, Ana Luiza Dallora Moraes, Abbas Cheddad, Peter Anderberg, Andreas Jakobsson, Johan Sanmartin Berglund

**Affiliations:** 1 Department of Health, Blekinge Institute of Technology, 371 41 Karlskrona, Sweden; 2 Institute of Computer Science, University of Tartu, Narva mnt 18, 51009 Tartu, Estonia; 3 Mathematical Statistic, Lund University, 221 00 Lund, SE Sweden

**Keywords:** Classification, Chronic obstructive pulmonary disease (COPD), Machine learning, Vowel segmentation, Prognostic markers, Biomarkers, Health care, Diagnostic markers, Predictive markers, Prognostic markers

## Abstract

Vowel-based voice analysis is gaining attention as a potential non-invasive tool for COPD classification, offering insights into phonatory function. The growing need for voice data has necessitated the adoption of various techniques, including segmentation, to augment existing datasets for training comprehensive Machine Learning (ML) modelsThis study aims to investigate the possible effects of segmentation of the utterance of vowel "a" on the performance of ML classifiers CatBoost (CB), Random Forest (RF), and Support Vector Machine (SVM). This research involves training individual ML models using three distinct dataset constructions: full-sequence, segment-wise, and group-wise, derived from the utterance of the vowel "a" which consists of 1058 recordings belonging to 48 participants. This approach comprehensively analyzes how each data categorization impacts the model's performance and results. A nested cross-validation (nCV) approach was implemented with grid search for hyperparameter optimization. This rigorous methodology was employed to minimize overfitting risks and maximize model performance. Compared to the full-sequence dataset, the findings indicate that the second segment yielded higher results within the four-segment category. Specifically, the CB model achieved superior accuracy, attaining 97.8% and 84.6% on the validation and test sets, respectively. The same category for the CB model also demonstrated the best balance regarding true positive rate (TPR) and true negative rate (TNR), making it the most clinically effective choice. These findings suggest that time-sensitive properties in vowel production are important for COPD classification and that segmentation can aid in capturing these properties. Despite these promising results, the dataset size and demographic homogeneity limit generalizability, highlighting areas for future research.

*Trial registration* The study is registered on clinicaltrials.gov with ID: NCT06160674.

## Introduction

Chronic Obstructive Pulmonary Disease (COPD) is a progressive respiratory disorder characterized by a gradual diminution of airflow and lung tissue deterioration. It has emerged as a significant global health concern, ranking as the third leading cause of mortality and morbidity worldwide^1,2^. In 2015, approximately 174 million individuals were diagnosed with COPD, with an estimated 3.2 million deaths, likely underestimated due to high underdiagnosis rates^3,4^. COPD is not only related to pulmonary problems; it is known to have systemic effects^5^, meaning that having COPD may lead to the malfunction in other organs. Even though the main evaluation is based on spirometry and Computerized Tomography (CT)^6^, recent research has investigated the possibility of using systemic effects to support decision-making processes^7,8^. COPD is known to affect voice production^9,10^, which has increased interest in investigating the potential utilization of various vocal parameters as decision-support cues for COPD diagnosis^11,12^. The underlying premise is to use machine learning (ML) algorithms to extract latent information embedded within vocal characteristics to support the decision-making process for early diagnosis.

Speech processing encompasses various techniques, including noise reduction methods to enhance signal clarity^13^, feature extraction approaches to analyze vocal characteristics^14^, and strategies like additive white noise to improve model robustness under different SNR levels^15^. The process of extracting information from voice entails the mathematical computation of attributes associated with individual voice samples, commonly referred to as voice, vocal features, and vocal biomarkers^16^. These features can be derived from time, frequency, and spectral representations of the raw voice recordings, such as baseline acoustic features (BLA), Jitter and Shimmer, and Mel Frequency Cepstral Coefficients (MFCC), which are techniques that emanate from voice recognition and provide a foundation for research in the field of voice-based decision support systems^16,17^. The characteristics of voices differ widely. Different voice-affecting disorders influence different voice characteristics. For example, Parkinson's disease tends to affect the vowel "a" phonation; on the other hand, Alzheimer's disease influences free speech^17^. Even individual differences in voice types are highlighted in the literature referring to the unliterary and dynamic characteristic of voice production^18^.

The evaluation of Artificial Intelligence (AI) for performing medical tasks is underway across various fields of practice. Using voice recordings, vocal features, and ML to diagnose disorders that affect the voice is a growing area of interest among researchers^19–21^. The idea is to extract information from voice recordings and let ML assess patterns that can be used for clinical purposes, such as detection, classification, and monitoring, to support decision-making processes^22–24^. ML is an active research area involving the systematic development of algorithms for better performance to mimic humankind's abilities based on the collected data^25,26^. Additionally, the performance of ML in complex data analyses is another factor for the increased usage in clinical research^27–31^. However, the common denominator for ML-based experiments is the demand for data, which in some cases might be challenging to work with and require additional techniques to train ML models on small datasets^32^. There are several techniques employed to expand the voice datasets to make it possible to train ML algorithms on more data, such as the collection of several recordings at the same time^33–36^, using windowing with some degree of overlap to create several feature vectors from one single recording^37–39^, or dividing the recording into time frames and treating each frame as a new recording^40–42^. However, these methods are applied mostly on long speech recordings, with very few studies investigating their efficacy for vowel recordings. Since vowel production exhibits dynamic characteristics^43,44^, segmentation techniques tailored for vowels may have a different impact on ML performance compared to their application in continuous speech. Furthermore, shorter time frames may capture more stationary characteristics of voice signals^45^, which could influence classifier performance in ways not yet fully explored. While these methods are widely employed, it is essential to explore their potential impact on the performance of ML classifier, particularly in the context of vowel-based analysis.

This article investigates whether time frame-wise differences in the utterance of the vowel 'a' collected from Swedish-speaking individuals affect the binary classification performance of ML algorithms CB, RF, and SVM in distinguishing between COPD and non-COPD voices. The aim is to apply segmentation to the utterance of the vowel 'a' to assess performance differences across individual and grouped datasets compared to the full sequence of recordings and analyze the classification results from a clinical perspective, exploring whether segmentation enables a more refined analysis of disease-related vocal characteristics and enhances the diagnostic relevance of voice-based features. The potential contributions of this study include:

- Introducing or refining a segmentation method could provide insights into how analyzing smaller segments of vocal data, rather than the entire recordings, impacts classification performance.
- A comparison of the CB, RF, and SVM on the performance effects of segmented vs. full sequence data offers valuable knowledge on which algorithms are best suited for segmentation-based extended datasets.
- By focusing on time frame-wise differences, the study may uncover whether there is a time sensitivity in recordings critical for COPD classification, which could help in developing more advanced and precise speech analysis models and signal processing frameworks.
- By analyzing time frame-wise differences in vowel utterances, the study may enhance the accuracy of COPD and non-COPD voice classification, leading to more effective early diagnosis tools using ML algorithms.

## Results

This section provides an analysis of the effects of segmentation of the vowel "a" utterance for binary classification performance (COPD vs. No COPD) from an experiment involving three machine learning classifiers: CB, RF, and SVM. Confusion matrix results are provided to compare the overall accuracy metrics for segment-based and group-wise results, a clinically relevant perspective on performance. Additionally, the Receiver Operating Characteristic (ROC) curves provides a comparation of the ML results between using full-sequence dataset and segmented dataset that achieved the best performance.

### Experimental results

**Fig. 1:**
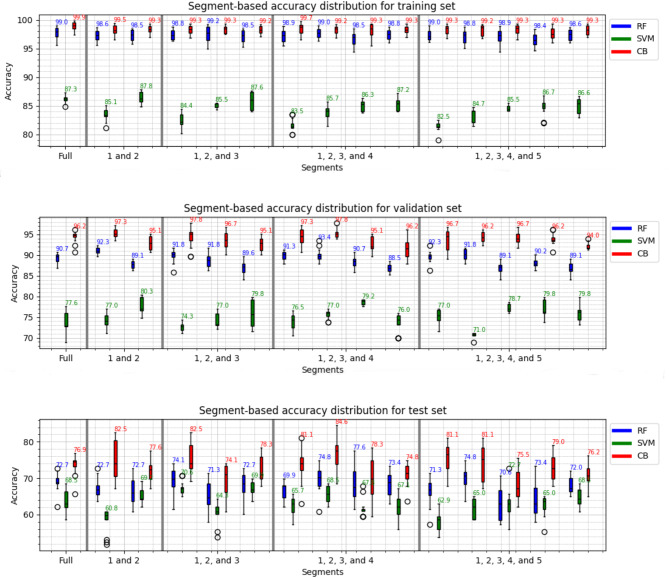
Segment-wise accuracy results show the maximum accuracy achieved for each segment with three different ML classifiers for the training, validation, and test sets, where the whole sequence is divided into several segment combinations, starting from full sequence to 5 different segments.

The experiment that forms the basis of Fig. 1 yielded 15 distinct results for each machine learning classifier. The different combinations of nested cross-validation (nCV), starting from 2X2 to 5X5, have generated 16 results for each performance metric: precision, recall, accuracy, and F1 score, associated with each segment in five different categories, starting from the full sequence and ending with five equally divided segments of the same recording.

Figure 1 illustrates the distribution of the accuracy results and the highest accuracy achieved for the training validation and test datasets, displayed at the top of each boxplot and for each dataset. The classifiers CB, RF, and SVM ranked from highest to lowest based on their performance: training set accuracies of 99.9%, 99.2%, 87.8%; validation set accuracies of 97.8%, 93.4%, and 80.3%; and the test set accuracies of 84.6%, 77.6%, and 72.7%, respectively. The highest accuracies were measured mostly in segment-based results, with the exception of the CB classifier in the training set, where the highest accuracy of 99.9% was achieved in the full segment. Compared with the full-sequence results, an overall improvement in performance metrics was noted for segment-based results. However, the performance improvement in the test set seems to come with a cost of increased variance, which is not just across different segments but also when comparing the segmented results to the full sequences for both the validation and test sets, while the training set results look stable. Moreover, when examining the results of the validation and the test sets on a pairwise and segment-wise basis, each classifier exhibits a unique trend slope. For the validation set, the trend lines for the CB and RF classifiers exhibit similar patterns, whereas the SVM displays a divergent trend. Conversely, the test set results demonstrate a greater variation among the trend lines of all three classifiers compared to the training and validation sets. Another interesting observation is that the ensemble learning-based classifiers CB and RF show better performance and achieve higher average accuracy mostly in the first half of the recordings when divided into two halves, with performance metrics summed and averaged separately for each half. However, SVM follows the opposite trend by having the highest accuracies in the second half of the recordings for the validation set and test set.

Table 1 presents all the validation and test set results for all segments and all performance metrics associated with each ML classifier. The analysis of the table reveals distinct performance metrics for CB, RF, and SVM models across various all categories and metrics within both validation and test sets. Specifically, the CB model performed consistently better than the other models across most metrics, achieving its peak precision of 97.6%, the highest accuracy of 97.8%, and F1 scores of 97.8%, in the 3-segment category and a recall of 98.2%, in 4-segment category during validation. The RF model shows strong performance, especially in the 4-segment category for a precision of 93.0% and recall of 93.8%, and it reaches its best performance with an accuracy of 93.4% and F1 score of 93.3% within the 4-segment category. On the other hand, SVM lags behind with its best precision of 79.7%, recall of 79.6%, accuracy of 80.3%, and F1 score of 79.6%, all occurring in the 2-segment category, underlining its comparative underperformance. When shifting focus to the performance on the test set, CB's supremacy persists with the highest precision of 84.7%, a top recall rate of 84.8%, and a leading accuracy and F1 scores of 84.6% and 84.6%, respectively, all in the 4-segment category. RF maintained its highest test performance with precision, recall, accuracy, and F1-score of 77.2%, 77.8%, 77.6%, and 77.6%, respectively, in the 4-segment category. Conversely, SVM falls behind results in the test set similar to the validation set, with its highest precision of 73.7%, recall of 73.2%, accuracy of 73.7%, and F1 score of 72.6%, respectively, in the 5-segment categories. However, when comparing the performance drop between the highest validation and test set results for each classifier, the SVM classifier demonstrates a higher degree of generalizability. It shows the smallest decrease across all metrics, with drops of 6.0% in precision, 6.4% in recall, 7.6% in accuracy, and 7.0% in F1-score. The CB classifier takes second place with precision, recall, accuracy, and F1-score of 12.9%, 13.1%, 13.2%, and 13.2%, respectively. The RF classifier suffers the highest performance drop with precision, recall, accuracy, and F1-score of 15.3%, 16.0%, 15.8%, and 15.7%, respectively. The result indicates an increased level of possible overfitting within a performance drop comparison between the full sequence results of validation end test sets, where performance drop for full segment occurs for SVM, 7.7%, 7.7%, 8.1%, 8.3%, and CB, 17.4%, 19.1%, 19.3%, 19.3%, and lastly for RF, 14.7%, 17.2%, 18.0%, 18.2%, on the performance metrics precision, recall, accuracy, and F1-score, respectively.

**Table 1:** Validation and test set scores for three different ML classifiers using four different performance metrics for full sequence and each segment.

Validation set metrics															
Model	Full	2 Seg		3 Seg			4 Seg				5 Seg				
		1	2	1	2	3	1	2	3	4	1	2	3	4	5
Precision (%)															
CB	95.8	97.0	94.7	**97.6**	96.4	94.8	97.0	97.5	94.7	96.0	96.5	96.0	96.8	95.8	93.6
RF	90.5	91.9	88.6	91.3	91.3	89.1	90.8	**93.0**	90.2	88.0	91.9	91.5	88.6	89.7	88.5
SVM	76.9	76.5	**79.7**	73.8	76.4	79.1	75.8	76.4	78.8	75.2	76.5	70.3	78.1	79.1	79.1
Recall (%)															
CB	96.6	97.5	95.4	97.9	97.0	95.0	97.5	**98.2**	95.4	96.2	96.8	96.4	96.4	96.6	94.1
RF	90.7	92.7	88.9	92.0	92.0	89.8	91.6	**93.8**	90.9	88.4	92.7	92.0	89.1	90.7	89.3
SVM	76.7	75.5	**79.6**	72.3	76.3	**79.6**	76.2	75.9	79.4	74.9	75.5	70.8	77.5	79.4	79.4
Accuracy (%)															
CB	96.2	97.3	95.1	**97.8**	96.7	95.1	97.3	**97.8**	95.1	96.2	96.7	96.2	96.7	96.2	94.0
RF	90.7	92.4	89.1	91.8	91.8	89.6	91.3	**93.4**	90.7	88.5	92.4	91.8	89.1	90.2	89.1
SVM	77.6	77.1	**80.3**	74.3	77.1	79.8	76.5	77.1	79.2	76.0	77.1	71.0	78.7	79.8	79.8
F1_Score (%)															
CB	96.1	97.2	95.0	**97.8**	96.6	94.9	97.2	**97.8**	95.0	96.1	96.6	96.1	96.6	96.1	93.8
RF	90.5	92.2	88.8	91.6	91.6	89.4	91.1	**93.3**	90.5	88.2	92.2	91.6	88.8	90.0	88.8
SVM	76.8	75.8	**79.6**	72.7	76.3	79.3	75.9	76.1	78.8	75.0	75.8	70.4	77.7	79.2	79.2
Test set metrics															
Precision (%)															
CB	78.4	82.6	78.1	82.7	75.8	78.9	81.2	**84.7**	78.3	77.3	81.2	82.0	75.9	79.3	78.3
RF	75.8	72.8	73.5	75.2	71.3	74.1	70.2	75.1	**77.7**	74.8	71.7	75.5	70.6	74.3	72.6
SVM	69.2	61.6	71.3	70.7	66.2	72.3	65.7	69.0	69.4	68.9	62.8	65.3	**73.7**	66.5	70.6
Recall (%)															
CB	77.5	82.7	77.9	82.3	74.6	78.7	81.3	**84.8**	78.4	75.4	80.1	81.5	75.8	79.3	76.9
RF	73.5	72.8	73.1	74.6	71.3	73.3	70.2	75.0	**77.8**	73.7	71.6	75.2	70.6	73.8	72.4
SVM	68.9	61.3	69.9	70.8	65.0	70.7	65.7	68.8	68.4	67.8	62.8	65.2	**73.2**	65.6	69.0
Accuracy (%)															
CB	76.9	82.5	77.6	82.5	74.1	78.3	81.1	**84.6**	78.3	74.8	81.1	81.1	75.5	79.2	76.2
RF	72.7	72.7	72.7	74.1	71.3	72.7	69.9	74.8	**77.6**	73.4	71.3	74.8	70.6	73.4	72.0
SVM	68.5	60.8	69.2	70.6	64.3	69.9	65.7	68.5	67.8	67.1	62.9	65.0	**72.7**	65.0	68.5
F1_Score (%)															
CB	76.8	82.5	77.6	82.4	74.1	78.3	81.1	**84.6**	78.3	74.7	81.0	81.1	75.5	79.0	76.0
RF	72.3	72.7	72.7	74.0	71.3	72.7	69.9	74.8	**77.6**	73.4	71.3	74.8	70.6	73.4	72.0
SVM	68.5	60.8	68.9	70.6	64.3	69.6	65.7	68.5	67.6	66.8	62.8	65.0	**72.6**	64.7	68.4

The highest performance is shown in bold.

CB outperforms RF and SVM across all categories, achieving the highest accuracy of 97.8%, precision of 97.6%, recall of 98.2%, and F1-score of 97.8% in the validation set. In the test set, CB maintains its lead with an accuracy of 84.6%. RF follows but shows the highest performance drop, indicating potential overfitting. SVM, while the lowest-performing model, exhibits the smallest drop in performance, suggesting better generalizability.

**Fig. 2:**
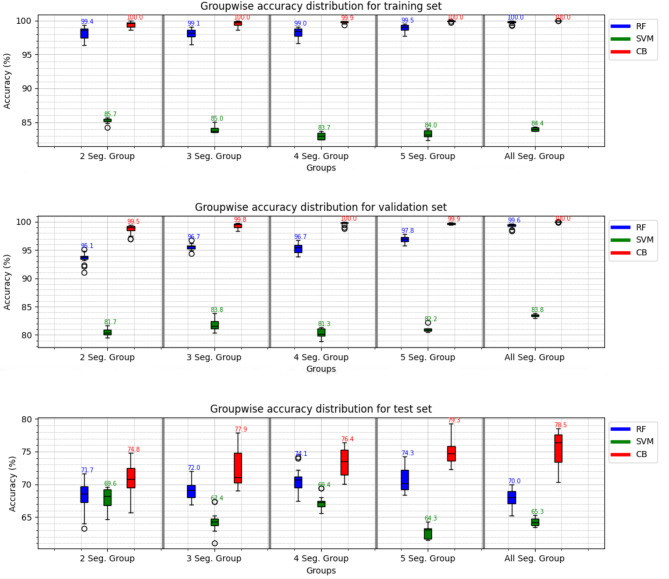
Group-wise accuracy results show the max accuracy achieved in each group with three different ML classifiers for training validation and test sets, where segments in Fig. 1 are merged into a dataset to create respective groups, first from two to five and then all segments together.

Figure 2 presents the results when the feature vectors in each segment-based data merged into one dataset to create an expanded dataset with a factor of the number of segments in the specific group. Additionally, all-segment group is created by merging all feature vectors from all segment-based groups and the full sequence together, creating a dataset with a factor of 15 from one single recording if the recordings are divided into five segments as highest. The CB classifier performs with the highest accuracy of 100% on the training set and validation set overall groups. RF classifier follows CB with a small decrease in training and validation set accuracy of 99.5% and around 97.0% on average, respectively. However, SVM falls behind with an accuracy of 84.0% and 82.0% average for training and validation set results, respectively. Test set results do not change the ranking where CB, RF, and SVM classifiers are placed in chronological order from highest to lowest performance with accuracies of 79.3% in 5 segment group, 74.3% 5 segment group, and 69.6% in 2 segment group, respectively. In a comparison between Figs. 1 and 2, it is clear that variation in performance decreases when the grouped dataset is used. On the other hand, the higher accuracies achieved in Table 1 results are not presented in the group-wise results. Regardless of which dataset is employed, the results indicate a higher performance than using features extracted from a full sequence of vowel "a" utterance regarding the performance accuracy.

Group-wise results of all three classifiers for validation and test set are presented in Table 2. The results suggest that the CB performs the best in all metrics with a score of 100% in two segment groups, 4 and all-segment groups in validation sets. The RF classifier gives the second-best performance with the highest score of 99.6% in all metrics in the all-segment group for the validation set. The SVM classifiers take the last position in performance for the validation set with the highest scores of 83.3%, 83.5%, 83.8%, and 83.3%, Precision, Recall, Accuracy, and F1-score, respectively, in the all-segment group. The group-wise results for the test set suggest that the CB classifier is the best-performing one with scores of 80.6%, 78.9%, 79.3%, and 79.0%, in precision, recall, accuracy, and F1 score, respectively, in the 5-segment group with the exception of precision that reaches the highest score in the all-segment group. RF reaches its highest precision, recall, accuracy, and F1-score of 74.2%, 74.3%, 74.3%, and 74.2% in the 5-segment group for the test set, respectively. SVM falls behind with precision, recall, accuracy, and F1 scores of 70.0%, 69.9%, 69.6%, and 69.6%, in the 2-segment group for the test set, respectively. From the generalizability point of view, regarding performance drops between unseen data and unseen participants, presented by validation and test set, respectively. SVM, CB, and RF follow the chronological order regarding the lowest performance drop in precision, recall, accuracy, and F1 score by 13.3%, 15.8%, 13.9%, 13.7%, and 19.4%, 21.1%, 20.7%, 21.0%, and lastly 25.4%, 25.3%, 25.3%, 25.4%, respectively. That indicates that the models trained on the group-wise expanded dataset generate a higher performance drop than those results presented in Table 1.

**Table 2:** Validation and test set scores for three different ML classifiers using four different performance metrics for each group.

Validation set metrics					
Model	2 Seg. group	3 Seg. group	4 Seg. group	5 Seg. group	All Seg. group
Precision (%)					
CB	99.3	99.8	**100.0**	99.9	**100.0**
RF	94.6	96.2	96.6	97.8	**99.6**
SVM	80.8	82.8	81.1	81.8	**83.3**
Recall (%)					
CB	99.6	99.9	**100.0**	99.9	**100.0**
RF	95.2	97.2	96.7	97.7	**99.6**
SVM	80.5	83.7	80.8	81.7	**83.5**
Accuracy (%)					
CB	99.5	99.8	**100.0**	99.9	**100.0**
RF	95.1	96.7	96.7	97.8	**99.6**
SVM	81.7	83.8	81.3	82.2	**83.8**
F1_Score (%)					
CB	99.4	99.8	**100.0**	99.9	**100.0**
RF	94.9	96.6	96.6	97.8	**99.6**
SVM	80.7	83.2	80.8	81.8	**83.3**
Test set metrics					
Precision (%)					
CB	75.5	78.0	76.6	80.0	**80.6**
RF	72.2	72.1	74.1	**74.2**	69.9
SVM	**70.0**	68.5	70.0	65.5	66.6
Recall (%)					
CB	75.2	78.0	76.2	**78.9**	77.9
RF	72.0	71.8	74.0	**74.3**	69.9
SVM	**69.9**	67.9	69.7	64.7	65.9
Accuracy (%)					
CB	74.8	77.9	76.4	**79.3**	78.5
RF	71.7	72.0	74.1	**74.3**	70.0
SVM	**69.6**	67.4	69.4	64.3	65.3
F1_Score (%)					
CB	74.8	77.9	76.2	**79.0**	78.0
RF	71.7	71.8	74.0	**74.2**	69.9
SVM	**69.6**	67.2	69.4	64.3	65.1

The highest performance is shown in bold.

CB remains the strongest model, achieving perfect scores of 100% in multiple validation set groups. RF follows with a peak accuracy of 99.6%, while SVM lags behind, with its best accuracy at 83.8%. In the test set, CB maintains the highest accuracy of 79.3%, followed by RF (74.3%) and SVM (69.6%). Performance drops are more pronounced in group-wise datasets, with SVM showing the least decline, further supporting its generalizability.

The performance results underscored the robustness of CB and superior performance across all dataset configurations, with RF following closely behind. Although SVM showed notable generalizability, it generally trailed behind the other models. In addition to the performance metrics, Table 3 presents the confusion matrix results, which offer a clinical perspective of the ML model’s performance. Here, the recall metric, also known as the true positive rate (TPR), is highlighted as it indicates the model’s ability to correctly identify positive cases, a clinically relevant factor in clinical applications. The confusion matrix results show that while the best performance order is preserved as CB, RF, and SVM, the highest TPR is aligned with the highest accuracies in the performance metrics results in Tables 1 and 2. The best TPR of 75/75 and 72/75 for CB and RF, respectively, were achieved in the 4 Seg. category, and for SVM, the highest TPR of 59/75 has been achieved in the 3 Seg. category for the validation set. The confusion matrix test set results also show a deviation compared to performance metrics results in Tables 1 and 2, where the best TPR occurs in 4 Seg, Full, and 2 Seg. categories are 60/68, 61/68, and 67/68 for CB, RF, and SVM, respectively. Group-wise confusion matrix results for the validation set are aligned for CB and RF with the performance metrics results in Tables 1 and 2, which are TPR of 1124/1124 and 1119/1124, respectively. However, SVM diverges and reaches its highest TPR of 177/213 in 3 Seg. group. For the test set results, CB and RF reached their highest TPR of 112/136 and 107/136 in the 2 Seg. Groups and SVM have a TPR of 160/204 in 3 Seg. Group. Almost all high TPRs are achieved using segment-wise datasets or group-wise datasets with only one exception, where the RF classifier performed the best TPR in the full segment category.

**Table 3:** Confusion matrix results for validation and test sets gained from the ML models showed the highest average accuracy in each dataset configuration, where the best result is bolded for each ML classifier. The highest performance is shown in bold.

Segment-wise											
Validation set											
		Full		2 Seg		3 Seg		4 Seg		5 Seg	
		Predicted ( +)	Predicted (−)	Predicted ( +)	Predicted (−)	Predicted ( +)	Predicted (−)	Predicted ( +)	Predicted (−)	Predicted ( +)	Predicted (−)
CB	Actual( +)	74	1	74	1	74	1	**75**	**0**	73	2
	Actual(−)	6	102	4	104	3	105	**4**	**104**	4	104
RF	Actual( +)	68	7	71	4	70	5	**72**	**3**	71	4
	Actual(−)	10	98	10	98	10	98	**9**	**99**	10	98
SVM	Actual( +)	54	21	56	19	**59**	**16**	56	19	58	17
	Actual(−)	20	80	17	91	**21**	**87**	19	89	20	80
Test set											
CB	Actual( +)	60	8	58	10	53	15	**60**	**8**	61	7
	Actual(−)	25	50	15	60	10	65	**14**	**61**	20	55
RF	Actual( +)	**61**	**7**	55	13	57	11	55	13	56	12
	Actual(−)	**32**	**43**	26	49	26	49	19	56	24	51
SVM	Actual( +)	52	16	**57**	**11**	50	18	54	14	56	12
	Actual(−)	29	46	**33**	**42**	24	51	25	50	27	48

Group-wise											
Validation set											
		2 Seg. group		3 Seg. group		4 Seg. group		5 Seg. group		All Seg. group	
CB	Actual( +)	142	0	213	0	**309**	**0**	387	0	**1124**	**0**
	Actual(−)	2	222	1	335	**0**	**357**	1	527	**0**	**1626**
RF	Actual( +)	136	6	211	2	298	11	376	11	**1119**	**5**
	Actual(−)	12	212	16	320	13	410	9	519	**5**	**1616**
SVM	Actual( +)	107	35	**177**	**36**	229	80	305	82	910	214
	Actual(−)	32	192	**53**	**283**	57	366	81	447	230	1391
Test set											
CB	Actual( +)	**112**	**24**	165	93	208	64	240	100	663	357
	Actual(−)	**48**	**102**	56	169	111	189	48	327	104	1021
RF	Actual( +)	**107**	**29**	136	68	195	77	252	88	702	318
	Actual(−)	**52**	**98**	52	173	71	229	96	279	326	799
SVM	Actual( +)	103	33	**160**	**44**	208	64	243	97	787	233
	Actual(−)	54	96	**96**	**129**	11	189	158	217	511	614

CB and RF achieve the highest recall (TPR) in the 4-segment validation category, while SVM’s best TPR is in the 3-segment group. In the test set, CB and RF peak in the 4-segment and full-segment groups, whereas SVM’s best TPR is in the 2-segment group. The results confirm CB’s superior classification performance, RF’s strong but slightly overfitting nature, and SVM’s generalizability despite lower overall accuracy.

**Fig. 3:**
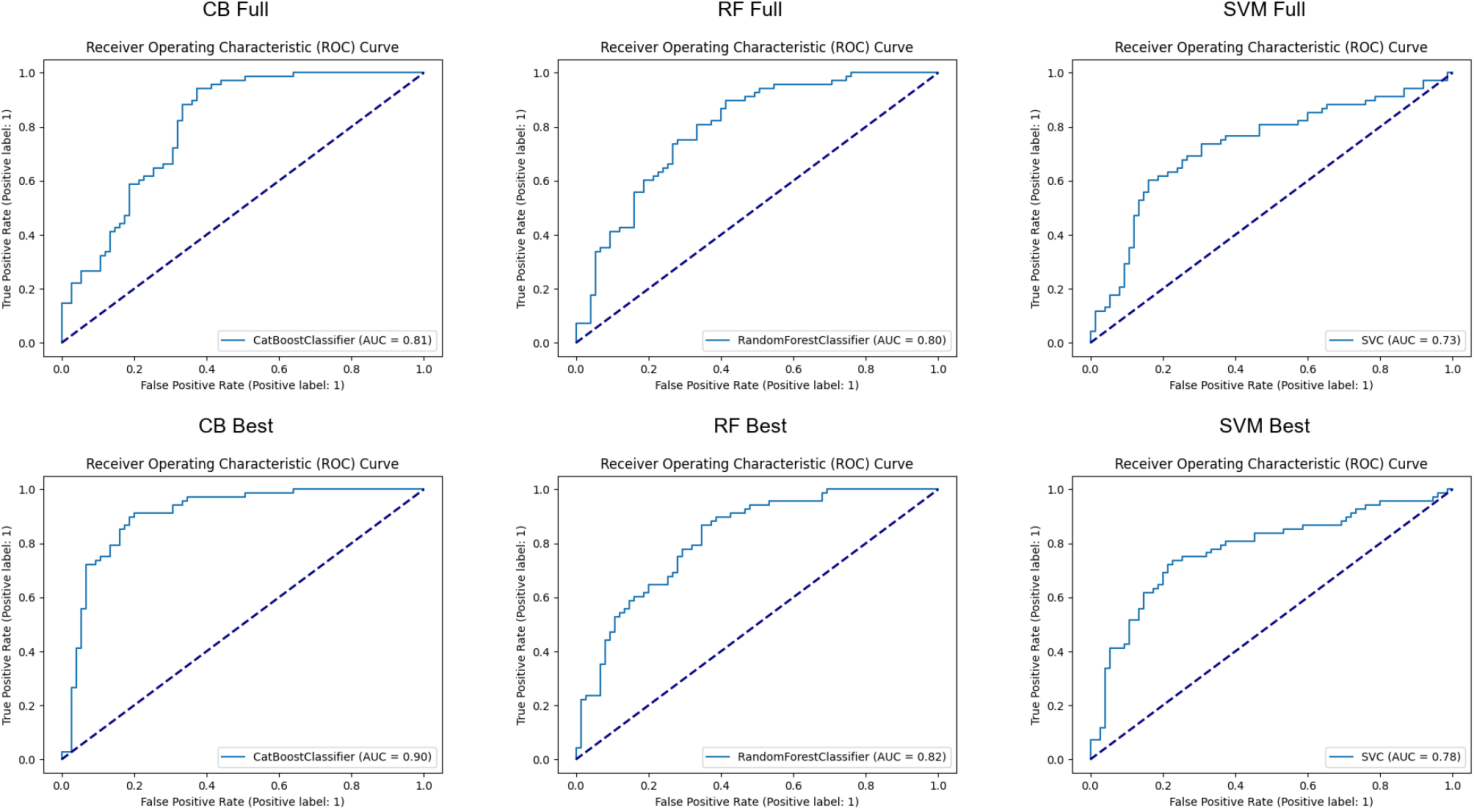
ROC curves for ML models trained on the full voice sequence and segmented voice data, where the best performance was achieved.

Examining the performance improvement between the models trained on the full voice sequence and the best-performing segmented version, using the ROC curves presented in Fig. 3, reveal notable differences across classifiers. The CB classifier, which initially achieved an AUC of 0.81 when trained on the full sequence, improved to 0.90 when using the segmented data, demonstrating the most substantial performance gain. Similarly, the RF classifier showed a slight improvement from 0.80 to 0.82, while the SVM model increased from 0.73 to 0.78. Similar to the previous metrics presented in Tables 1,2, and 3, the ROC curves also indicate that CB outperforms the other models across all decision thresholds. The steeper initial rise of the ROC curve for CB Best suggests a stronger separation of classes compared to the full-sequence approach.

## Discussion

This study employed a segmentation method to observe how different segments and groups of segments of the utterance of the vowel "a" affect the binary classification performance of three classifiers, RF, SVM, and CB, for the classification of COPD and non-COPD voices. This study uniquely contributes to the field by specifically examining the impact of segmentation on ML performance, a topic not fully explored in prior research. It provides new insights into how different uses of time frames affect model outcomes, offering a deeper understanding of their potential to enhance performance consequently generating academic engagement with this research direction. The key findings in this study are as follows:

- Using segments individually or in groups mostly generated higher performance metric scores as compared to using the features extracted from the full sequence.
- Segmenting data increases classification accuracy. However, this increase comes with a cost of higher variation in classification performance.
- Expanding the dataset by merging the segments in groups reduces the variance observed in segment-wise classification performance but never reaches the highest performance achieved in the segments.
- The features extracted from the first half of the recordings show a higher classification performance on average than the second half of the recordings for tree-based algorithms CB and RF. However, the distance-based SVM shows fluctuation.

In general, segmentation proves to be an effective method for creating datasets composed of BLA and MFCC features extracted from time frames of the vowel "a" recordings. This might be connected to the voice signals depicting stationary characteristics in shorter time frames^45^. However, this approach appears to benefit tree-based algorithms, such as RF and CB, more than distance-based algorithms, like SVM. The SVM classifier did not show as much improvement as RF and CB. The fluctuations in SVM performance across the datasets, compared to CB and RF, may be due to its sensitivity to feature distribution. Unlike CB and RF, which adaptively handle non-linear patterns, SVM’s hyperparameter sensitivity may cause inconsistencies across the datasets, even with grid search optimization. The ability of CB and RF to weigh features differently may contribute to their more consistent performance. Considering the increased variation, a key challenge with this method is identifying the correct segment within the recording. In this study, the second segment out of a four-segment category achieved the highest performance, with validation and test set accuracies of 97.8% and 84.7%, respectively, using the CB classifier. This result is further supported from a clinical standpoint by the confusion matrix, which shows a TPR of 100% (75/75) on the validation set and 88.2% (60/68) on the test set. When combined with other performance metrics for the same segment, the high recall suggests that this approach may help ensure that most moderate-level COPD cases are identified^46,47^. This is particularly relevant given that the COPD cohort in this study primarily consists of participants in moderate stages, with an average ratio of Forced Expiratory Volume in 1 s (FEV1) and Forced Vital Capacity (FVC) around 0.61%. From a clinical perspective, another critical consideration is maintaining a balance between TPR and False Positive Rate (FPR) to avoid unnecessary overdiagnosis^6,47^. In this regard, CB seems to achieve a reasonable balance in both the validation and test sets within the four-segment category, providing reliable performance without compromising clinical relevance. Group-wise results in Table 2 and Fig. 2 show a smoothing effect on the results with decreased variation. However, the greater performance drop between the validation and test sets indicates a higher degree of possible overfitting to the validation data^48,49^. This problem might be mitigated by increasing participant variability, including factors like balanced age and health status, increased sample size, using regularization techniques, and data augmentation in future studies. Additionally, from a clinical point of view, expanding a dataset based on grouping the small time frames does not seem to support real-world scenarios, as represented by the test set results in Table 3. The best TPR results in the group-wise analysis do not reflect better performance than segment-wise or full-sequence results.

The results suggest that certain time frames within the recordings exhibit more deterministic properties, which could enhance classification performance. This situation may be due to the more pronounced differences in certain voice feature characteristics, as reported in previous studies^50,51^. Specifically, the second segment in the four-segment group appears to have performed the best, which could be attributed to the unique temporal or acoustic properties that distinguish it from other segments. It would be valuable to investigate why the second segment yielded superior results, whether it’s due to a specific change in vocalization patterns or a shift in the underlying physiological state of the subjects. This also reflects the dynamic characteristics of the vowel production^43,44^. However, the findings indicate that different ML models, such as CB, RF, and SVM, excel in different segments of the recordings. This implies that the signal processing steps should be optimized to match the characteristics of the chosen model. Consequently, employing a fixed recording duration, as seen in other data collection methods^52^, or data extracted from a fixed frame of a signal^53^ may not fully leverage the performance potential of models for COPD classification. By integrating segmentation strategies with ML models, future diagnostic tools could achieve higher accuracy and robustness, enabling earlier and more precise identification of COPD. These findings could inform the development of clinical workflows that leverage vocal biomarkers for screening, monitoring, and early intervention.

The computational demands of this study were substantial due to 21 dataset configurations, 3 ML models, 3 hyperparameter options, and a 5 × 5 nCV framework, resulting in 625 + training runs per model and a total training period exceeding 3 months. While the primary objective was to assess the impact of segmentation on model performance, an important observation was that grouping segments into new datasets to expand them significantly increased training time and memory usage across all models, in proportion to the number of segments grouped. However, segmentation itself did not increase memory usage or the time required to train the models compared to using the full sequence of the recording and remained 220 MB–33.8 s, 204 Mb–40.3 s, and194 MB–384.0 s for the best performing CB, RF, and SVM, respectively, as the total number of data points remained unchanged. This suggests that while segmentation enhances model performance, dataset expansion strategies may introduce computational trade-offs that should be considered in practical ML applications for voice analysis in COPD classification.

With respect to all the strengths in this study, such as employing well-known ML methods and voice features, applying regularization and nested cross-validation to minimize overfitting and increase generalizability, it is essential to acknowledge the limitations of the present study. This study is based on a dataset collected from a small cohort of individuals who primarily speak the dialect of southern Sweden. This limited sample size and linguistic homogeneity may restrict the generalizability of the findings. Applying the findings to other populations or dialects may present challenges due to differences in speech patterns, vocal characteristics, and demographic factors. Variations in accent, pronunciation, and language use across regions or dialects could impact the model's ability to generalize. Additionally, limited representation of certain age and gender groups could introduce biases and affect the generalizability of the findings. While the study provides valuable insights, future work should focus on increasing the dataset size and ensuring a more balanced demographic distribution to improve robustness and applicability across diverse populations. However, this limitation may provide a more concentrated analysis that could be more challenging to achieve with a widespread dataset. Another limitation is that the analysis is constrained to CB, RF, and SVM classifiers in the context of COPD, may not be applicable to other models, such as artificial neural networks, or to other voice-affecting disorders, such as Parkinson's disease^54^. Additionally, the study focuses solely on sustained vowel ”a”, which may not fully capture articulatory and phonatory variations present in other vowels, consonant–vowel pairs, or connected speech. Expanding the analysis to include these elements, along with deep learning approaches, could offer a more comprehensive understanding of respiratory-phonatory coordination in real-world conditions. Future studies should analyze statistical differences more thoroughly to better understand the statistical significance of feature variations. Furthermore, the cross-sectional design of the study does not provide longitudinal analysis for understanding changes and trends over time, which might be another area worth investigating alongside the investigation of the computational efficiency of different segmentation strategies aspect in more detail, particularly in the context of real-time applications or resource-constrained environments in future research.

The variability in segment-wise results suggests that alternative techniques, such as wavelet transformation^55^, may capture the temporal characteristics of voice without requiring segmentation. This approach could eliminate the need to identify the most suitable segment for classification. Future research should explore this potential and investigate further optimization of voice assessment systems for COPD detection. Additionally, addressing this study's limitations by testing larger datasets, exploring multilingual applications, and incorporating longitudinal data will help to enhance the reliability of voice-based diagnostic tools.

## Conclusion

These findings suggest that time-sensitive properties in vowel production are important for COPD classification, and that segmentation can help capture these properties. However, expanding the dataset by grouping segments does not necessarily improve performance, especially in real-world scenarios. Potential future applications of a voice assessment system for COPD include aiding early diagnosis through vocal feature analysis, supporting disease monitoring, and facilitating personalized management strategies. However, the clinical utility of such a system would depend on further validation in larger, real-world datasets. Additionally, if demonstrated to be effective, such a system could serve as a decision-support tool for clinicians, potentially improving diagnostic accuracy and optimizing resource allocation in healthcare settings.

## Methods

### General description

**Fig. 4:**
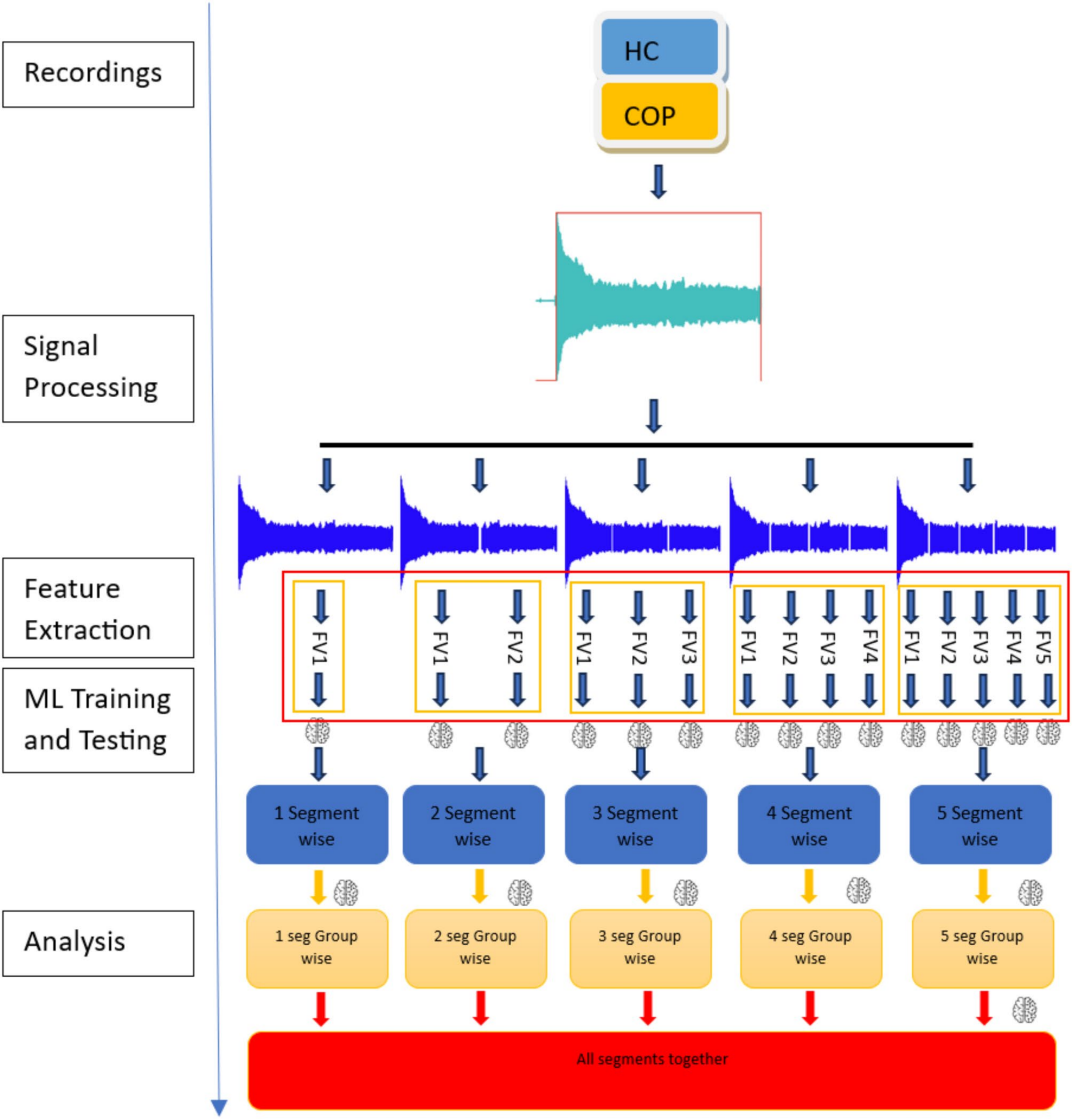
Chronological overview of the workflow. FV stands for feature vectors extracted from each sample.

This study conducts experiments on the utterance of entire vowel "a" recordings by dividing the entire recording into several subsegments and comparing the binary classification performance of three ML models: CB, RF, and SVM segment-wise and group-wise. Segment-wise datasets involve training models on smaller time frames of the full recording, while group-wise datasets involve merging multiple segments into a single dataset, increasing the number of samples available for training. The models were trained using nCV on different combinations in the number of inner and outer folds. Figure 4 illustrates the workflow and segmentation proceeds of this study.

### Data acquisition

The dataset employed in this study was created from Swedish utterances of the vowel "a" recordings collected through a mobile application *VoiceDiagnostic*, from a pool of research clinic participants who were recruited at the Blekinge Institute of Technology (BTH) in Sweden. Sixty-eight participants provided 1246 recordings in total. 30 COPD (16 female and 14 male) and 38 Healthy Control (HC) group (20 female and 18 male) made 436 and 810, respectively. Participants with COPD had an average FEV1/FVC of 0.61%, with a standard deviation of 0.12%. The voices recorded by VoiceDiagnostic, which is an application compatible with Android and iPhone, allows participants to make two types of recordings: one single utterance of the vowel "a" with the maximum possible duration and a scripted speech provided in the application. However, this study analyses only utterances of the vowel "a" recordings because the sustained vowel "a" is widely recognized as providing a controlled, reliable measure of vocal fold function and acoustic stability^56–58^. Participants were enrolled after a brief introduction to the study was given by a nurse with experience in research and after meeting the first author, who provided deeper information about the study. Participants were instructed to record in a quiet environment, free from background noise. Each recording was manually checked by the author to confirm it was free from any unwanted sounds. The participant's integrity and safety against the risk of data leakage were ensured by anonymizing the data and securing both physical and digital data in secure cabinets and safe databases. Each participant was assigned a unique ID to ensure that no personal information was used, and all data were anonymized to protect privacy, especially given the sensitivity of voice data. The study was approved by the Swedish ethics review authority in Umeå (DNR: 2020-01045) and followed the principles of the Declaration of Helsinki. All participants signed a written informed consent form that allows the collection of voice samples, health data, and sociodemographic information during a six-month period. The recruitment was based on the inclusion and exclusion criteria given below:

### Inclusion criteria

- COPD group
  Participants starting from 18 years old or older who have a COPD diagnosis, with access and proficiency to use a smartphone.
- HC group
  Participants starting from 18 years old and older who do not declaring that they have a voice-affecting disorder diagnosis, i.e., no disorder listed in the categories' nonlaryngeal aerodigestive disorders affecting voice', 'neurological disorders affecting voice', and 'systematic conditions affecting voice' in the Classification Manual for Voice Disorders^54^, with access and proficiency to use a smartphone.

### Exclusion criteria

- COPD group
  Participants younger than 18 years old with a voice-affecting disorder other than COPD, or declare no access or proficiency to smartphone use.
- HC group
  Participants younger than 18 years old with a voice-affecting disorder or declaring no access or proficiency to use a smartphone.

### Data preparation and feature extraction

The voice recordings were checked and standardized on parameters regarding sampling frequency and silence-free sequences. 44.1kHZ frequency was common for all voice recordings, and the silence part, which is usually common at the beginning and end of the vowel recordings, was dispatched from the recordings using a basic moving average filter and adaptive threshold that standardized the process for each recording^50^. The silence-free voice signals were divided equally into several one-dimensional segments from 2 to 5, separately based on the total duration of the voice part of the recording as visualized in Fig. 4. These choices were made to comply with the constrains of potential mobile implementations such as limited processing power, memory, and battery life. By including the original full recording, 15 different feature vectors were calculated for each recording for five distinct experiments, as it is shown in Fig. 1.

The feature vectors contained 107 parameters related to some demographics (Age, and Gender), baseline acoustics (BLA) (Duration, 2 Fundamental Frequency measures, Harmonic to noise ratio, 5 Jitter measures, 5 Shimmer measures, 4 mean formant measures, and 4 median formant measures) and Mel-Frequency Cepstral Coefficients (MFCC) (Standard deviation and mean of first 13 MFCCs and first and second derivative of them), where a detailed description of the features extracted from the vowel "a" are described in a previous study performed on the same dataset^50^. These features were chosen for their promising performance in earlier studies on different types of COPD voices^50,51^. Jitter and Shimmer measure frequency and amplitude perturbations, respectively, reflecting vocal fold stability. Increased values are linked to respiratory and laryngeal impairments, including COPD. MFCCs capture spectral properties of speech and are widely used in pathological voice classification due to their effectiveness in modeling vocal tract characteristics^56,59,60^. The features were extracted using Praat (Parselmouth) and Librosa libraries using Python.

### Experimentation with machine learning

In order to mitigate the imbalance problems that affect the performance, the data set was balanced by matching the feature vectors based on gender and age. That resulted in a dataset containing 1058 recordings belonging to 48 participants (24 females and 24 males), which was used in ML experiments. A subset of 25% (12/48 participants and 143/1058 recordings) of participants (12 participants, 6 females, and 6 males, 143 recordings) based on matched age with ± 6.7 years old standard deviation with an average age of 73.2 years old were isolated for the test dataset for the evaluation of ML models on unseen data for the training set. Further, the remaining 75% (36/48 participants and 917/1058 recordings) of participants (36 participants, 18 females, and 18 males) with ± 6.5 years old standard deviation with an average age of 75, 1 years old were further divided into two sub-datasets, 80% (732/917 recordings) training dataset and 20% (183/917 recordings) validation dataset corresponding to 732 and 183 recordings, respectively. This data distribution was done to observe ML models' learning and classification performance on data collected from the same participant from different time stamps.

Nested cross-validation nCV, also known as double k-fold cross-validation, is a method utilized to mitigate the overfitting^61,62^. This technique combines traditional k-fold cross-validation in two stages: an outer loop and an inner loop. In this method, the outer loop divides the data into k folds, and for each fold, the inner loop performs cross-validation on the training data to tune the hyperparameters. This process helps ensure that the model is not overfitting to the training data and provides a more reliable estimate of model performance. It was employed for the training of CB, RF, and SVM classifiers^63–65^, which were suggested for their ability to handle tabular data with relatively small sample sizes while effectively capturing non-linear patterns. These classifiers have demonstrated strong performance in similar datasets across multiple studies^19,66,67^, including a previous investigation focused on COPD classification^50^. Additionally, the limited sample size in this study constrained the feasibility of testing larger models, as proven by an initial LSTM test. The LSTM model exhibited high overfitting due to insufficient data, ultimately leading to the decision to abandon the pursuit of larger models. Hyperparameter optimization was performed using a grid search within the inner loop of nested cross-validation to choose the best performing model. The optimized hyperparameters that provided the best performance with specific nCV combinations on the models are as follows:

- CB: 5X2nCV, ('depth': 4, 'iterations': 300, 'l2_leaf_reg': 5, 'learning rate': 0.1).
- RF: 4X4nCV, ('max depth: None, 'min samples split': 5, 'n estimators': 200).
- SVM: 5X3nCV, ('C': 1, 'degree': 2, 'kernel': 'linear').

### Analysis

Alongside the performance measures of accuracy, F1-score, precision, recall metrics and ROC curves, the confusion matrix was used to elucidate the results from a clinical perspective. The observed results were presented in the form of graphs and tables.

## Data Availability

The raw recordings cannot be made available due to ethical and general data protection regulations. However, an anonymized version of the dataset after the pre-processing of voice, generated during the present study will be made available from the corresponding author's institution upon reasonable request. The code for repeating the experiments can be found on GitHub: https://github.com/AIITPlanet/Code/blob/main/Analysis_RF_SVM_CB_Nested_ForPartitionsToExcel.py.
